# Changes in age at last birth and its determinants in India

**DOI:** 10.1038/s41598-023-37370-z

**Published:** 2023-06-27

**Authors:** Mayank Singh, Chander Shekhar, Neha Shri

**Affiliations:** 1 Department of Fertility & Social Demography, International Institute for Population Sciences (IIPS), Mumbai, 400088 India; 2 Department of Survey Research and Data Analytics, International Institute for Population Sciences (IIPS), Mumbai, 400088 India

**Keywords:** Health care, Health policy, Behavioural methods

## Abstract

In recent years, developing and developed countries are witnessing delayed childbearing among women contributing to the overall decline in fertility rates. The age at which a woman has her last child impacts maternal and child health, especially in a country with high maternal and perinatal mortality rates. This study aims to investigate the trends of age at the last birth among Indian women and to identify the potential factors contributing towards higher maternal age. The present study uses the data from five consecutive rounds (1992–1993, 1998–1999, 2004–2005, 2015–2016, and 2019–2021) of the National Family Health Survey (NFHS). We have used descriptive statistics, bivariate, Cox proportional hazard regression analysis, multiple classification analysis (MCA), Kaplan–Meier curve, life table survival analysis, hierarchical clustered heat map, multivariate decomposition analysis (MDA) and geospatial mapping to fulfill the objective of the study. Results show that the proportion of women with age at last birth before reaching the age of 30 years was less than half (nearly 35%) during NFHS-I while during NFHS-V proportion becomes more than half and reaches 64.3% among 40–49 years women. Within three decades (1992–2021) there has been a decline of 15.8% in median age at last birth among women aged 40–49 years. Additionally, the highest percentage decline in predicted mean age at last birth was noted among individuals from rural area (10.7%, 3.3 years), Hindu religion (10.8%, 3.3 years), poor wealth quantile (12.5%, 4.0 years) and those with mass media exposure (10.6%, 3.2 years) from NFHS-I (1992–1993) to NFHS-V (2019–2021). Although there exists the need to delay age at first childbirth, the age at last childbirth also plays an important role in women’s and child health status. Hence, it is important to address the healthcare needs of those delaying their childbirth.

## Introduction

Biological aging among women and its association with females' fertility has always garnered plenty of attention worldwide^1,2^. In recent years, developing and developed countries are witnessing delayed childbearing among women contributing to the overall decline in fertility rates. Since the first and last birth age defines a women’s realized reproductive period, the last birth denotes the end of their reproduction despite being fecund^3^. The declining age at the last birth is an indication of the onset of the demographic transition from high to low fertility in a country^4^. The actual ending of childbearing in a woman occurs much before the end of reproductive age, even in the absence of contraceptives^4^. The age at which a woman has their last child impacts maternal and child health, especially in a country with high maternal and perinatal mortality rates. The evidence indicates that during the 1970s, the age at first birth was deficient, and women started childbearing at very early ages with the average age at last birth ranging from 39 to 42 years, resulting in higher fertility levels^5,6^. However, in recent years, most nations have seen a decrease in the number of young women giving birth, which has been driven by increased educational attainment, family planning methods, and behavioural changes^7,8^. For instance, women from England and Wales have their first child 5 years later than in the 1970s^9^. A report published by United Nations states that childbearing at a later age reduces the number of births in countries with high maternal mortality due to premature deaths among women of reproductive ages^10^. Arguments existing in the literature are controversial regarding the effect of late childbearing on offspring and maternal health. Some researchers believe that late childbirth is associated with postponed aging, good health and increased longevity^1,11^. Studies have also highlighted more common adverse pregnancy outcomes among women aged 35 and above^12,13^. Numerous previous studies have shown adverse pregnancy outcomes among women of older ages. For example, a study conducted in the United States found that women aged 35 years were twice more likely to have a miscarriage, and women over the age of 40 had a 2.4 times higher chance of miscarriage than women aged less than 35 years^14^. The age of 40 and older is in itself identified as an independent risk factor for gestational diabetes, placental abruption, perinatal mortality^14^, and hypertensive complications^7^. On the other hand, infant deaths were more pronounced among women aged 50 years or older^15^.

Previous studies have reported varying ages at the last birth among different populations^3^. The variation in the age at the last birth is due to random processes^16^. Fertility behavior is influenced by social and cultural factors as well as individual and cultural variances that exist in communities and result in various fertility behaviors^17^. A literature review conducted in 2014 reported that parents considered having a job, a stable income, and decent housing to be crucial factors when deciding whether or not to have children^18^. This variation could be due to heterogeneity across women in the timing of infecundity^19^. Even if all women had the same fertility rates, by chance, each woman would have a variable waiting period for conception as well as a unique history of miscarriage and infant mortality.

Although the factors determining the variability in age at the last birth are not well-studied, researchers believe that age at the last birth is influenced by social factors and the choice of individuals in the form of family limitations^20^. Trends analysis from the past shows that older maternal age at the last birth is associated with a high level of fertility and mortality^21^. Over the previous three decades, several developed countries have witnessed an increase in child-bearing ages, mainly attributable to changing women’s roles and expectations^22,23^. An increasing proportion of women from developing countries are also postponing marriage^24^. According to UN report published in 2015, since childbearing among women is getting delayed in pursuit of career and other personal objectives, the issue of advanced maternal age becomes more apparent in women’s health^25^. Although the arguments available from the literature make it hard to define the specific age for pregnancy outcomes, the effects of increasing age on pregnancy outcomes can’t be ignored.

Similarly, several problems and detrimental effects associated with advanced maternal age and childbearing are highlighted^26,27^. Some studies in the past have documented the impact of advanced maternal age and delayed childbearing on perinatal outcomes among women^28,29^. Studies have reported a higher incidence of pretersm birth, intrauterine growth restriction, fetal malformation, and neonatal deaths among children born to older mothers^30,31^. Further, women having a pregnancy at older ages are at higher risk of pregnancy complications, including maternal mortality and severe morbidities^32^.

In a country like India, marriage drives the age at sexual debut and childbirth, and a girl is expected to bear a child soon after the marriage. Findings from the Indian demographic health survey indicated that 7.9% of adolescent girls aged 15–19 were already mothers by the time of the survey. A wide variety of research is available in the context of the cause or consequence of fertility rates and sex ratio. Still, very little attention has been paid to the timing of childbearing. Despite an enormous amount of literature on the ill effect of age at the last birth amongst mothers and infants, the timing of the completion of childbearing is a relatively less-explored area in the context of India, and the existing information is scarce. This study aims to investigate the pattern of age at last motherhood among Indian women and identify the potential factors contributing to changing maternal age at last birth nearly over four decades in the Indian settings.

## Methods

### Data source

The present study uses the cross-sectional, nationally representative data from five consecutive rounds of the National Family Health Survey (NFHS), (in 1992–1993, 1998–1999, 2004–2005, 2015–2016, and 2019–2021 resp.) conducted by the International Institute for Population Sciences under the guidance of the Ministry of Health and Family Welfare (MoHFW), Government of India. NFHS is a cross-sectional household survey among women aged 15–49 years. The NFHS provides a wide range of reliable estimates related to fertility, family planning practices, reproductive health, and maternal and child health at national and sub-national levels. The NFHS survey is a large-scale, multi-round, three-stage sampling design for the first three rounds of the survey and a two-stage stratified sampling design for the fourth and fifth rounds of the survey. NFHS has been conducted covering all the states and union territories of India since 1992 and utilizes the previous census as a sampling frame (except for the first round of NFHS; the sampling frame was taken from the 1981 census for most of the states) in each round of the survey. The number of women interviewed during the various round of NFHS was 89,777, 90,303, 124,385, 699,686, and 724,115 in consecutive survey rounds with the overall response rate of 96.1, 95.5, 94.5, 96.7, and 97 percent respectively^34–36^. Women aged 40–49 without a desire for more children were eligible for this study and the sample size at the consecutive rounds was 15,226, 16,202, 21,685, 139,079 and 148,655 in NFHS-I to NFHS-V respectively.

### Variable description

#### Outcome variable

Age at the last birth of eligible women is the variable of interest in this study. Currently married women aged 40–49 years, who have reported at least one birth, were drawn from the overall sample of women aged 15–49 years. Since, by the age of 40–49 years, there is a biological cessation of childbearing ages, the fertility rates are very low in those ages. Because more than 90% of women age 40–49 years completed their childbearing by 40 years of age. Women who expressed the desire for another birth were excluded from the analytical sample in each round of the survey as this may underestimate the age at the last birth if these women plan to continue reproducing^37^.

#### Predictor variables

The predictor variables considered in this study were region, residence, religion, caste, wealth index and education. Individual caste was clubbed together for other backward caste (OBC) and general categories for NFHS-1 as we did not have separate data for these two caste categories. From NFHS-2 onwards, individuals in OBC and other castes have been taken separately. Further, mass media exposure was coded as ‘Any’ if women responded other than “not at all or not” for the questions (i) Do you read a newspaper or magazine almost every day, at least once a week, less than once a week, or not at all? (ii) Do you listen to the radio almost every day, at least once a week, less than once a week, or not at all? (iii) Do you watch television almost every day, at least once a week, less than once a week, or not at all? (iv) Do you usually go to a cinema hall or theatre to see a movie at least once a month? And coded as No if not exposed to any type (newspaper or magazines, radio, television, cinema hall) of media. Prior relationship with husband means whether the woman was related to her husband in any way prior to her marriage. This variable was included on the pretext that cross-cousin or consanguineous marriage shows altogether a different childbearing pattern. Detailed information on the categorization of variables used in the study is tabulated below in Table 1.

**Table 1:** Description of variables included in the analysis.

Variables	Recode
Regions	Coded as 1 = east, 2 = west, 3 = north, 4 = south, 5 = central, 6 = north-east
Respondent’s level of education	Coded as 1 = illiterate, 2 = primary, 3 = secondary, 4 = higher
Caste	Coded as 1 = scheduled caste, 2 = scheduled tribe, 3 = OBC, 4 = others
Religion	Coded as 1 = Hindu, 2 = Muslims, 3 = Christians, 4 = others
Wealth index	Coded as 1 = poorest, 2 = poorer, 3 = middle, 4 = richer, 5 = richest
Mass media exposure	Coded as 1 = no, 2 = any
Place of residence	Coded as 1 = urban, 2 = rural
Age at first marriage (AFM)	Coded as 1 = less than 15 years, 2 = 15–18 years, 3 = more than or to equal 18 years
Age at first sex (AFS)	Coded as 1 = less than 15 years, 2 = 15–18 years, 3 = more than or to equal 18 years
Working status	Coded as 1 = no, 2 = yes
Previous parity	Coded as 1 = zero, 2 = 1–2 parity, 3 = 3–4 parity, 4 = 5 and more parity
Family structure	Coded as 1 = nuclear, 2 = non-nuclear
Contraceptive demand	Coded as 1 = unmet need, 2 = met need, 3 = no demand
Prior relationship with husband	Coded as 1 = no, 2 = yes

### Statistical analysis

Descriptive analysis was carried out to understand the distribution of background variables for the study samples. Bivariate analysis was carried out to see the distribution of respondents by age at the last birth at different exact ages and the median age at the last birth was calculated. Geospatial mapping was used to show the state-specific variation in predicted mean age at the last birth for the last three survey rounds. Additionally, failure life table estimates were used to understand the true probability of non-occurrence of the last birth. In this article, we applied the Kaplan–Meier survival plot approach to obtain the probability of women who had not given their last birth by a certain age among eligible women.

We also used Cox Proportional hazard regression analysis to look at the impact of the time of the last birth from a multivariable perspective. The Cox model is expressed by the hazard function denoted by h(t). Briefly, the hazard function can be discussed for the current study as the risk of last birth at time t. The hazard model can be written as follows:

$${\text{ln}}\left( {{\text{h}}\left( {\text{t}} \right)/{\text{h}}_{0} \left( {\text{t}} \right)} \right) = \left( {{\text{b}}_{{1}} {\text{x}}_{{1}} + {\text{b}}_{{2}} {\text{x}}_{{2}} + ... + {\text{b}}_{{\text{p}}} {\text{x}}_{{\text{p}}} } \right)$$

where h(t) indicates the expected hazard at time t, and h_0_(t) is the baseline hazard when all predictors equal zero. The variables Xi’s are the predictor variables, and bi’s indicate the coefficients associated with the covariates. In such a model, the outcome variable is the risk of hazard of experiencing the event (last birth). The hazard ratio for each independent variable represents the likelihood of experiencing the event for a particular group compared with the reference group. Furthermore, the predicted mean age at the last birth by various socioeconomic and demographic characteristics was also calculated with the help of Multiple Classification Analysis. The advantage of the MCA convergence model was that we can estimate the values of the reference category of the dependent variable, which was not possible in the Cox proportional hazard model or simple linear regression analyses.

To comprehend the factors influencing the change in the mean age at last birth among women aged 40–49 years from 1992 to 2021, a multivariate decomposition analysis was conducted. The primary objective of this analysis was to identify the sources responsible for the shift in the average age at last birth over the past three decades. This analysis involved breaking down the overall change in age at the last birth into two components: the change resulting from differences in the characteristics of the women surveyed (endowment) and the change attributable to variations in the impact of these characteristics (coefficient) between surveys.

All the analysis was carried out using Stata statistical software version 16.1 (StataCorp, College Station, TX), and graphical presentations were done in Arc-GIS and Origin Pro version 9.9.

### Limitations

First, the cross-sectional nature of data limits the understanding of the cultural transmission of reproductive behaviors. Second, some of the women expressed their desire for a child after the age of 40 years but those samples have been removed as their proportion was very low. Third, since we have taken women aged 40–49 years for calculating their age at last birth so it depicts a little older picture. Fourth, due to data availability issues and the creation of new states from erstwhile states, state-specific analysis was only conducted for the recent three survey rounds.

## Results

Table 2 illustrates the sample characteristics of women aged 40–49 years who already have given their last birth for each survey round by background characteristics. The percentage of respondents from the urban area has increased from 29.1% in NFHS-I to 34.4% in NFHS-V. The maximum share of women in all five survey rounds belonged to the Hindu religion and from the OBC caste. The proportion of women without formal education has decreased substantially from 67.0% in NFHS-I to 44.2% in NFHS-V. At the same time, the number of respondents with secondary and higher education increased more than twice from 1992–1993 to 2019–2021. Furthermore, there has been a consistent increase in the proportion of women with mass media exposure

**Table 2:** Sample characteristics of last birth among reproductive-aged women (40–49 years) by background characteristics.

Background characteristics	NFHS-I	NFHS-II	NFHS-III	NFHS-IV	NFHS-V
	Sample (%)	Sample (%)	Sample (%)	Sample (%)	Sample (%)
Age group					
40–44	8396 (55.1)	9211 (56.9)	12,326 (56.8)	70,776 (50.9)	72,713 (48.9)
45–49	6830 (44.9)	6991 (43.2)	9359 (43.2)	68,303 (49.1)	75,943 (51.1)
Regions					
East	2211 (14.5)	2794 (17.2)	3096 (14.3)	24,443 (17.6)	22,690 (15.3)
West	2285 (15)	2062 (12.7)	2919 (13.5)	12,093 (8.7)	16,200 (10.9)
North	3553 (23.3)	4020 (24.8)	4201 (19.4)	28,142 (20.2)	29,876 (20.1)
South	2915 (19.1)	2998 (18.5)	4375 (20.2)	20,686 (14.9)	27,704 (18.6)
Central	2806 (18.4)	2602 (16.1)	3806 (17.6)	35,789 (25.7)	32,266 (21.7)
Northeast	1456 (9.6)	1726 (10.7)	3288 (15.2)	17,926 (12.9)	19,919 (13.4)
Residence					
Urban	4424 (29.1)	4888 (30.2)	7398 (34.1)	50,152 (36.1)	51,133 (34.4)
Rural	10,802 (71)	11,315 (69.8)	14,287 (65.9)	88,927 (63.9)	97,522 (65.6)
Level of education					
No education	10,163 (67)	9432 (58.2)	12,186 (56.2)	69,349 (49.9)	65,709 (44.2)
Primary	2495 (16.4)	2838 (17.5)	3526 (16.3)	20,879 (15)	23,584 (15.9)
Secondary	2092 (13.8)	2849 (17.6)	4915 (22.7)	40,658 (29.2)	48,785 (32.8)
Higher	428 (2.8)	1078 (6.7)	1056 (4.9)	8194 (5.9)	10,577 (7.1)
Caste					
SC	1698 (11.2)	2755 (17.1)	3849 (18.4)	27,009 (20.2)	31,139 (22)
ST	1168 (7.7)	1110 (6.9)	1540 (7.4)	11,742 (8.8)	12,919 (9.1)
OBC	12,360 (81.2)	5418 (33.6)	8266 (39.5)	60,200 (45.1)	63,958 (45.1)
Others		6826 (42.4)	7257 (34.7)	34,626 (25.9)	33,876 (23.9)
Religion					
Hindu	12,561 (82.5)	13,348 (82.5)	17,866 (82.7)	114,527 (82.4)	123,425 (83.1)
Muslim	1576 (10.4)	1777 (11)	2372 (11)	16,082 (11.6)	16,831 (11.3)
Christian	469 (3.1)	491 (3)	601 (2.8)	3648 (2.6)	3880 (2.6)
Others	621 (4.1)	558 (3.5)	769 (3.6)	4754 (3.4)	4433 (3)
Wealth index					
Poorest	2110 (13.9)	2042 (12.6)	2128 (9.8)	24,854 (17.9)	28,368 (19.1)
Poorer	2111 (13.9)	2538 (15.7)	2872 (13.2)	28,545 (20.5)	31,612 (21.3)
Middle	2823 (18.5)	3202 (19.8)	3890 (17.9)	28,384 (20.4)	31,282 (21)
Richer	3782 (24.8)	3647 (22.5)	4959 (22.9)	27,625 (19.9)	29,340 (19.7)
Richest	4399 (28.9)	4774 (29.5)	7836 (36.1)	29,671 (21.3)	28,053 (18.9)
Mass media exposure					
No	7195 (47.3)	6308 (38.9)	5718 (26.4)	30,504 (21.9)	37,946 (25.5)
Any	8025 (52.7)	9894 (61.1)	15,967 (73.6)	108,575 (78.1)	110,709 (74.5)
Contraceptive demand					
Unmet need	965 (6.3)	795 (4.9)	1092 (5)	5784 (4.2)	5739 (3.9)
Met need	8413 (55.3)	10,462 (64.6)	14,342 (66.1)	88,908 (63.9)	115,782 (77.9)
No demand	5847 (38.4)	4945 (30.5)	6250 (28.8)	44,384 (31.9)	27,124 (18.3)
Age at first marriage					
< 15 Years	6270 (41.2)	5952 (36.8)	7156 (33)	26,812 (20.8)	26,308 (18.2)
15–18 Years	4739 (31.2)	5134 (31.7)	7041 (32.5)	38,097 (29.6)	43,911 (30.4)
≥ 18 Years	4204 (27.6)	5111 (31.6)	7487 (34.5)	63,843 (49.6)	74,232 (51.4)
Previous parity					
Zero	461 (3)	508 (3.1)	1103 (5.1)	11,328 (8.1)	12,799 (8.6)
1–2	3543 (23.3)	4934 (30.5)	8756 (40.4)	75,839 (54.5)	89,563 (60.3)
3–4	5160 (33.9)	5634 (34.8)	6900 (31.8)	35,851 (25.8)	34,468 (23.2)
5 +	6063 (39.8)	5126 (31.6)	4927 (22.7)	16,061 (11.6)	11,824 (8.0)
Age at first sex					
< 15 Years			4218 (20.6)	15,428 (12.7)	16,945 (12.1)
15–18 Years			7839 (38.3)	42,360 (34.7)	47,538 (33.9)
≥ 18 Years			8427 (41.1)	64,213 (52.6)	75,882 (54.1)
Household structure					
Nuclear			11,913 (54.9)	78,680 (56.6)	84,608 (56.9)
Non-nuclear			9772 (45.1)	60,399 (43.4)	64,047 (43.1)
Prior relationship to husband					
No				121,465 (87.4)	131,436 (88.4)
Yes				17,583 (12.6)	17,183 (11.6)
Total	15,226 (100)	16,202 (100)	21,685 (100)	139,079 (100)	148,655 (100)

Table 3 presents the distribution of women aged 40–49 years by their exact age at last and the median age at last birth. Our findings suggest that in earlier survey rounds, the majority of women (nearly 35%) aged 40–44 continued childbearing at later ages while in the recent survey round, almost 85% of women completed childbearing at the age of 35 years. Around 74% of the women aged 45–49 years completed their childbearing by the age of 40 years during NFHS-I whereas 89% of women of the same age completed their childbearing during NFHS-V. In the last three decades, India has experienced a 15.0%, 17.2%, and 15.8% decrement in median age at last childbearing among women aged 40–44 years, 45–49 years and 40–49 years respectively.

**Table 3:** Distribution of age at last birth by exact age in India over the period 1992–2021.

Percentage of women given their last birth by specific exact age and median age at last birth by current age 1992–2021										
Current age	NFHS-rounds	Percentage who gave last birth by exact age							Number of women	Median age at last birth
		30	35	36	37	38	39	40		
40–44	NFHS-I	38.8	65.0	69.0	72.0	75.0	77.4	79.3	8396	31.97
	NFHS-II	50.1	71.3	74.3	76.6	78.9	80.6	81.9	9211	29.98
	NFHS-III	60.8	82.8	85.4	88.1	90.4	92.1	93.2	12,326	28.2
	NFHS-IV	66.5	85.9	88.0	89.8	91.1	92.1	92.9	70,776	27.31
	NFHS-V	66.8	84.2	86.0	87.5	88.5	89.3	89.8	72,713	27.19
45–49	NFHS-I	31.2	55.6	59.5	63.3	67.5	70.6	73.6	6830	33.76
	NFHS-II	39.3	64.2	67.3	70.5	72.9	75.0	77.0	6991	31.92
	NFHS-III	54.3	77.2	80.9	84.0	86.7	89.4	91.1	9359	29.23
	NFHS-IV	60.7	81.9	84.6	86.8	88.8	90.5	91.8	68,303	28.22
	NFHS-V	61.9	81.3	83.7	85.5	86.9	88.1	88.9	75,943	27.97
40–49	NFHS-I	35.4	60.7	64.7	68.1	71.6	74.3	76.7	15,226	32.77
	NFHS-II	45.4	68.2	71.2	74.0	76.3	78.2	79.7	16,202	30.81
	NFHS-III	58.0	80.4	83.5	86.4	88.8	90.9	92.3	21,685	28.65
	NFHS-IV	63.7	83.9	86.3	88.4	90.0	91.3	92.3	139,079	27.75
	NFHS-V	64.3	82.7	84.8	86.5	87.7	88.6	89.3	148,655	27.59

**Figure 1:**
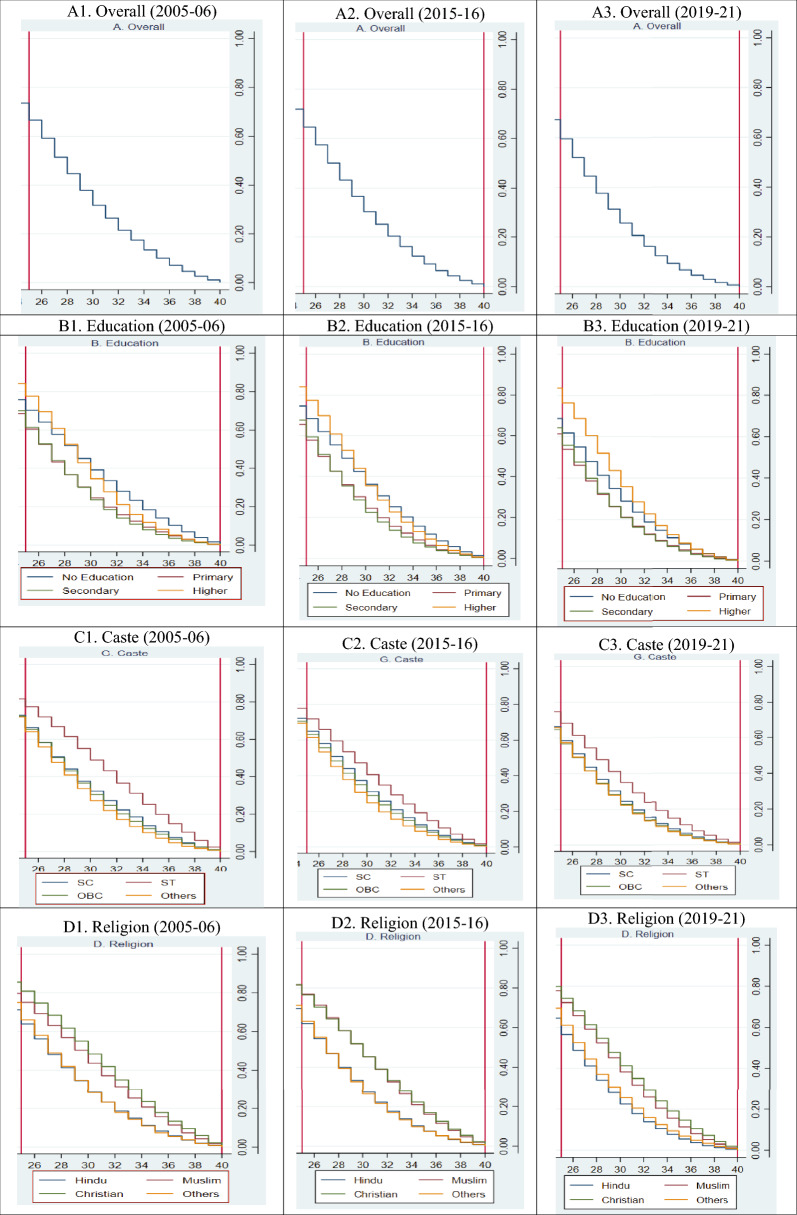

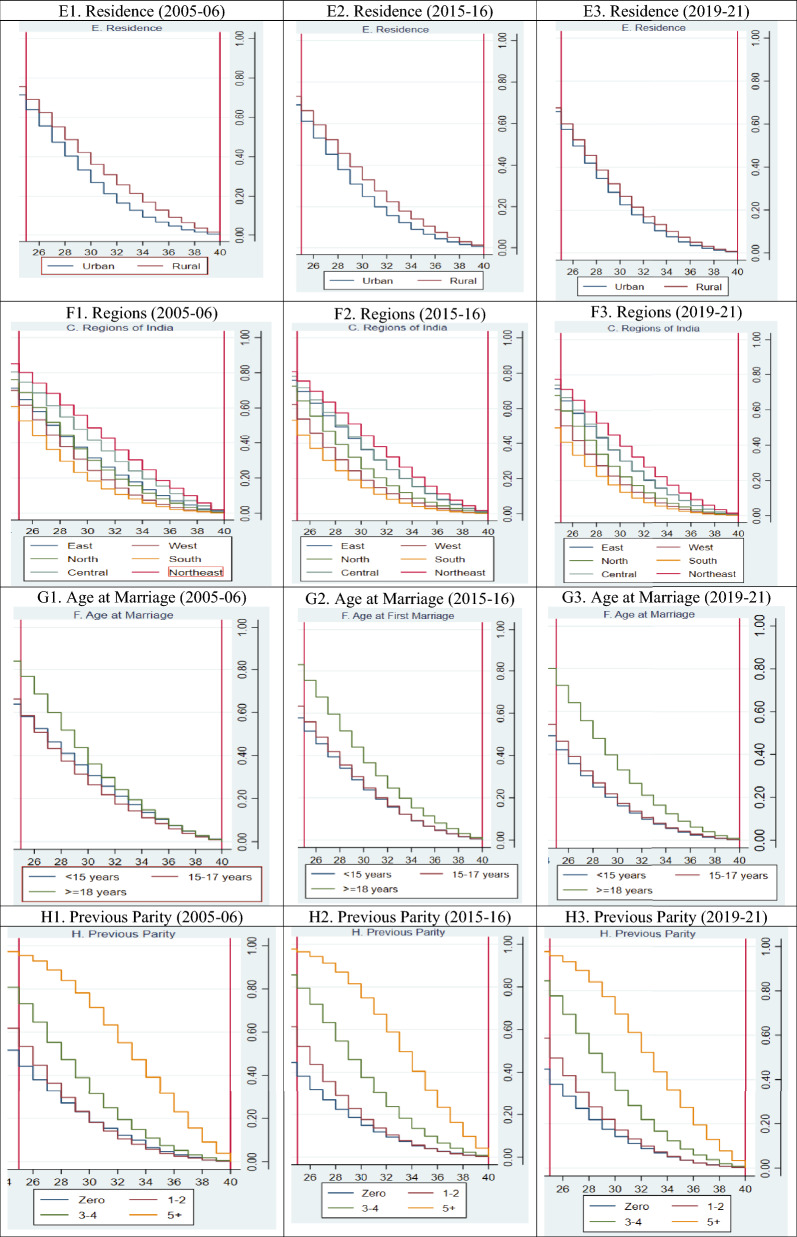
Kaplan–Meier failure estimates of age at last birth in India by background characteristics.

Kaplan Meier failure estimates of age at last birth in India by background factors are shown in Fig. 1. This figure shows that the overall age at the last birth has decreased over the years. By educational attainment, women with higher education have a greater probability of having higher age at last birth, and the age at last birth for women with no education is shifting downwards. Similar patterns in age at last birth can also be seen for religion, caste, and region.

The state-specific hierarchical clustered heat map is showed in Appendix Fig. A1 indicates the likelihood of the last birth not yet happened by exact age. The shorter the height of the dendogram between the two connecting states, the more similar the states are, whereas darker blue and darker red represent the higher and lower likelihood of the last birth not yet occurred by a specific age, respectively. During 2005–2006, Kerala showed a pattern similar to Maharashtra and Himachal Pradesh with Euclidian distance less than 0.02, however, during 2019–2021, Kerala, Maharashtra, Punjab, Rajasthan, Tamil Nadu and Uttarakhand showed the closest last birth pattern with Euclidian distance less than 0.02.

The Cox proportional hazard model predicting women's risks of last motherhood by various demographic characteristics is shown in Appendix Table B1. The table shows that factors such as region, education, caste, religion, wealth, mass media exposure, previous parity, and type of prior relationship with the husband are significantly associated with the age at the last birth. Among all associated factors, religion, age at first sex, and previous parity were the most prominent factors which showed larger variations in age at last birth. For instance, the women with previous parity of more than 4 have higher age at the last birth (AHR: 0.21: CI 0.21–0.22). Additionally, the age at last birth was higher among Muslim women (AHR 0.82*** [0.81,0.83]) in comparison to Hindus in model 3. Additionally, over the survey period, a significant decline in age at last birth has been observed in model 3 with AHR 1.50 (95% CI 1.47–1.53) in 2019–2021 in comparison to 1992–1993.

The variation in respondents' ages at the last birth was estimated using the predicted mean obtained from the multiple classification analysis (Table 4). The unadjusted values for any particular characteristics indicate the predicted mean age at the last birth when other socio-economic characteristics are not taken into consideration while the adjusted values indicate the predicted mean age at the last birth when all the considered socio-economic characteristics are held constant. In both the adjusted and unadjusted models, the mean age at the last birth has reduced substantially from the first round to the last survey round for all background characteristics. From the first to the last survey round, the Northern region experienced the greatest reduction in adjusted mean age at last birth (11.9%), followed by the Western region (11.5%). In all the rounds, the predicted mean age at last birth was found to be lower among those exposed to mass media, those who married before the age of 15 years, having age at first sex below 15 years, and the richest wealth-indexed women. To note, India shows a consistent decline in the mean age at last birth from the first to fifth survey round (30.6 in NFHS-1 to 27.4 in NFHS-V). The predicted median age at last birth did not vary much by caste and remained higher for women with higher parity.

**Table 4:** Multiple classification analysis of Predicted mean age at last birth among women aged 40–49 years by background characteristics, 1992–2021.

Variables	NFHS-I		NFHS-II		NFHS-III		NFHS-IV		NFHS-V	
	Unadjusted	Adjusted for factors and covariates	Unadjusted	Adjusted for factors and covariates	Unadjusted	Adjusted for factors and covariates	Unadjusted	Adjusted for factors and covariates	Unadjusted	Adjusted for factors and covariates
State region							Mean (95% CI)	Mean (95% CI)		
East (Ref)	31.2 [31.0 31.4]	30.7 [30.5 30.9]	29.6 [29.4 29.8]	29.4 [29.2 29.5]	28.3 [28.1 28.5]	28.4 [28.3 28.6]	28.9 [28.9 29.0]	28.4 [28.4 28.5]	28.1 [28.0 28.2]	27.7 [27.7 27.8]
West	29.5 [29.3 29.7]	30.3 [30.1 30.5]	28.1 [27.9 28.3]	29.1 [28.9 29.3]	27.3 [27.2 27.5]	28.3 [28.1 28.4]	26.4 [26.3 26.5]	27.9 [27.8 28.0]	26.2 [26.1 26.3]	26.8 [26.8 26.9]
North	30.5 [30.3 30.7]	31.0 [30.9 31.2]	29.4 [29.3 29.6]	29.7 [29.5 29.8]	28.4 [28.2 28.5]	28.6 [28.4 28.7]	27.8 [27.7 27.8]	28.4 [28.3 28.4]	27.2 [27.1 27.2]	27.3 [27.3 27.4]
South	29.1 [29.0 29.3]	29.9 [29.7 30.0]	27.6 [27.4 27.8]	28.5 [28.3 28.6]	26.3 [26.1 26.4]	27.7 [27.6 27.9]	25.3 [25.2 25.4]	27.5 [27.5 27.6]	25.1 [25.1 25.2]	26.6 [26.5 26.6]
Central	32.5 [32.3 32.7]	31.3 [31.1 31.4]	31.1 [30.9 31.3]	30.1 [29.9 30.3]	29.8 [29.6 30.0]	28.8 [28.7 29.0]	29.1 [29.0 29.1]	28.7 [28.6 28.7]	28.3 [28.2 28.3]	27.9 [27.8 27.9]
Northeast	31.4 [31.2 31.7]	30.5 [30.3 30.8]	30.9 [30.7 31.2]	29.4 [29.2 29.6]	30.8 [30.6 31.0]	29.2 [29.0 29.4]	30.0 [29.9 30.1]	29.0 [28.9 29.1]	29.3 [29.2 29.4]	28.0 [27.9 28.1]
Residence										
Urban	29.6 [29.4 29.7]	30.7 [30.6 30.8]	28.5 [28.3 28.6]	29.3 [29.2 29.4]	27.7 [27.6 27.8]	28.4 [28.3 28.5]	27.4 [27.3 27.4]	28.45 [28.4 28.5]	27.0 [26.9 27.0]	27.6 [27.5 27.6]
Rural	31.2 [31.1 31.3]	30.6 [30.5 30.7]	29.9 [29.8 30.0]	29.4 [29.3 29.5]	29.0 [28.9 29.1]	28.6 [28.5 28.7]	28.5 [28.4 28.5]	28.34 [28.31 28.4]	27.5 [27.5 27.5]	27.3 [27.3 27.4]
Highest education										
No education (Ref)	31.5 [31.3 31.6]	30.6 [30.5 30.7]	30.2 [30.1 30.3]	29.3 [29.1 29.4]	29.3 [29.2 29.4]	28.5 [28.4 28.6]	28.8 [28.8 28.9]	28.3 [28.3 28.3]	27.8 [27.7 27.8]	27.3 [27.2 27.3]
Primary	29.8 [29.6 30.0]	30.4 [30.2 30.5]	28.7 [28.5 28.9]	29.0 [28.8 29.1]	27.3 [27.2 27.5]	27.9 [27.7 28.0]	27.1 [27.1 27.2]	27.9 [27.8 27.9]	26.6 [26.6 26.7]	26.9 [26.9 27.0]
Secondary	28.6 [28.4 28.9]	30.6 [30.4 30.8]	27.9 [27.7 28.1]	29.3 [29.1 29.4]	27.3 [27.2 27.5]	28.3 [28.2 28.5]	27.1 [27.1 27.2]	28.3 [28.3 28.4]	26.8 [26.8 26.9]	27.4 [27.3 27.4]
Higher	29.7 [29.2 30.1]	32.4 [32.0 32.8]	29.1 [28.8 29.3]	31.1 [30.9 31.4]	29.1 [28.8 29.3]	30.3 [30.0 30.5]	29.2 [29.0 29.3]	30.6 [30.5 30.7]	29.1 [29.0 29.2]	29.7 [29.6 29.8]
Caste										
SC	31.7 [31.4 31.9]	30.8 [30.6 31.1]	29.7 [29.4 29.9]	29.3 [29.2 29.5]	28.3 [28.2 28.5]	28.4 [28.3 28.6]	28.2 [28.1 28.22]	28.5 [28.4 28.5]	27.2 [27.1 27.2]	27.4 [27.4 27.5]
ST	30.9 [30.6 31.1]	29.8 [29.6 30.1]	30.8 [30.5 31.1]	29.2 [28.9 29.4]	30.8 [30.6 31.0]	28.4 [28.2 28.6]	29.5 [29.5 29.6]	28.3 [28.2 28.4]	28.8 [28.7 28.8]	27.4 [27.3 27.5]
OBC	30.5 [30.4 30.6]	30.7 [30.7 30.8]	29.4 [29.2 29.5]	29.6 [29.5 29.7]	28.2 [28.1 28.3]	28.5 [28.4 28.7]	27.8 [27.8 27.9]	28.5 [28.4 28.5]	26.9 [26.9 27.0]	27.4 [27.4 27.5]
Others			29.0 [28.9 29.1]	29.3 [29.2 29.4]	27.8 [27.7 27.9]	28.5 [28.4 28.6]	27.5 [27.4 27.5]	28.2 [28.2 28.3]	27.0 [26.9 3]	27.3 [27.2 27.3]
Religion										
Hindu (Ref)	30.5 [30.4 30.6]	30.6 [30.5 30.7]	29.1 [29.0 29.2]	29.3 [29.2 29.3]	27.9 [27.8 28.0]	28.4 [28.3 28.4]	27.6 [27.6 27.7]	28.2 [28.2 28.3]	26.9 [26.9 26.9]	27.3 [27.2 27.3]
Muslim	32.5 [32.2 32.8]	31.0 [30.8 31.3]	31.2 [30.9 31.5]	29.7 [29.5 30.0]	30.0 [29.7 30.2]	28.9 [28.7 29.1]	30.1 [30.0 30.2]	29.1 [29.0 29.2]	29.1 [29.0 29.2]	28.3 [28.2 28.4]
Christian	30.5 [30.2 30.8]	31.1 [30.8 31.4]	30.7 [30.4 31.1	30.1 [29.8 30.5]	30.9 [30.7 31.2]	29.3 [29.0 29.5]	30.3 [30.2 30.4]	29.0 [28.9 29.1]	29.8 [29.7 29.9]	27.9 [27.8 28.0]
Others	29.9 [29.5 30.3]	30.1 [29.8 30.4]	29.0 [28.6 29.4]	29.2 [28.9 29.5]	28.3 [28.0 28.6]	28.2 [28.0 28.5]	27.8 [27.7 27.9]	28.2 [28.1 28.3]	27.5 [27.4 27.6]	27.2 [27.1 27.3]
Wealth index										
Poorest (Ref)	33.0 [32.7 33.2]	32.0 [31.7 32.1]	31.4 [31.2 31.6]	30.3 [30.1 30.6]	31.2 [30.9 31.4]	29.9 [29.7 30.1]	31.0 [30.9 31.1]	29.8 [29.7 29.9]	29.6 [29.6 29.7]	28.4 [28.4 28.5]
Poor	32.3 [32.0 32.5]	31.6 [31.4 31.8]	30.6 [30.4 30.8]	29.9 [29.7 30.1]	29.9 [29.7 30.1]	29.1 [28.9 29.3]	28.9 [28.9 29.0]	28.8 [28.7 28.8]	27.8 [27.7 27.9]	27.7 [27.6 27.7]
Middle	31.1 [30.9 31.3]	31.0 [30.8 31.1]	29.9 [29.7 30.1]	29.6 [29.4 29.8]	29.0 [28.6 29.0]	28.7 [28.5 28.8]	27.5 [27.5 27.6]	28.2 [28.1 28.3]	26.6 [26.5 26.6]	27.2 [27.2 27.3]
Richer	30.3 [30.1 30.5]	30.4 [30.2 30.5]	29.1 [28.9 29.2]	29.2 [29.1 29.4]	27.9 [27.7 28.0]	28.2 [28.1 28.4]	26.8 [26.7 26.8]	27.9 [27.8 27.9]	26.2 [26.2 26.3]	26.9 [26.9 27.0]
Richest	28.9 [28.8 29.1]	29.7 [29.5 29.9]	28.1 [27.9 28.2]	28.7 [28.6 28.9]	27.3 [27.2 27.4]	27.9 [27.8 28.0]	26.7 [26.7 26.8]	27.6 [27.5 27.6]	26.6 [26.6 26.7]	26.8 [26.7 26.8]
Mass media exposure										
No (Ref)	32.0 [31.8 32.1]	30.8 [30.7 30.9]	30.8 [30.7 31.0]	29.6 [29.4 29.7]	30.2 [30.1 30.4]	28.7 [28.6 28.9]	30.3 [30.2 30.4]	28.8 [28.8 28.9]	28.7 [28.6 28.7]	27.6 [27.6 27.7]
Any	29.7 [29.6 29.8]	30.5 [30.4 30.6]	28.6 [28.5 28.7]	29.3 [29.2 29.4]	28.0 [27.9 28.1]	28.4 [28.4 28.5]	27.4 [27.4 27.5]	28.2 [28.2 28.3]	26.8 [26.8 26.9]	27.3 [27.3 27.3]
Age at first marriage										
< 15 Years	31.0 [30.8 31.1]	29.4 [29.2 29.5]	29.6 [29.4 29.7]	27.9 [27.8 28.1]	27.6 [27.5 27.8]	28.3 [28.1 28.5]	26.3 [26.3 26.4]	27.0 [27.0 27.1]	25.0 [24.9 25.1]	25.8 [25.8 25.9]
15–17 Years	30.2 [30.0 30.3]	29.9 [29.8 30.1]	28.8 [28.6 28.9]	28.5 [28.4 28.7]	27.5 [27.4 27.6]	27.7 [27.5 27.9]	27.1 [27.0 27.1]	27.2 [27.1 27.3]	25.8 [25.8 25.9]	26.0 [26.0 26.1]
≥ 18 Years	30.8 [30.6 30.9]	32.4 [32.3 32.5]	29.7 [29.6 29.9]	31.1 [31.0 31.2]	29.5 [29.4 29.6]	29.1 [28.9 29.3]	29.5 [29.4 29.5]	29.5 [29.4 29.5]	28.9 [28.8 28.9]	28.6 [28.5 28.6]
Contraceptive demand										
Unmet need	36.2 [35.9 36.5]	34.6 [34.3 34.8]	34.7 [34.4 35.1]	33.0 [32.8 33.3]	33.0 [32.7 33.3]	30.8 [30.6 31.1]	32.0 [31.9 32.1]	30.5 [30.3 30.6]	29.8 [29.6 29.9]	28.8 [28.7 28.9]
Met need	29.7 [29.6 29.8]	30.0. [29.9 30.1]	28.4 [28.3 28.5]	28.7 [28.6 28.8]	27.5 [27.4 27.6]	28.0 [27.9 28.1]	27.4 [27.4 27.5]	28.0 [8.0 28.0]	27.0 [26.9 27.1]	27.2 [27.2 27.2]
No demand	31.2 [31.0 31.3]	31.0 [30.9 31.1]	30.6 [30.4 30.7]	30.2 [30.0 30.3]	29.5 [29.4 29.7]	29.2 [29.1 29.3]	28.7 [28.7 28.8]	28.8 [28.7 28.8]	28.0 [28.0 28.1]	27.8 [27.7 27.8]
Previous parity										
Zero	24.4 [24.0 24.8]	23.8 [23.4 24.2]	25.0 [24.6 25.4]	23.7 [23.3 24.0]	25.4 [25.1 25.7]	24.1 [23.9 24.4]	24.5 [24.4 24.6]	24.1 [24.0 24.2]	24.5 [24.4 24.6]	23.7 [23.7 23.8]
1–2	27.4 [27.3 27.5]	27.2 [27.1 27.4]	26.7 [26.6 26.8]	26.5 [26.4 26.6]	26.4 [26.3 26.5]	26.4 [26.3 26.4]	26.3 [26.3 26.3]	26.6 [26.6 26.6]	26.1 [26.1 26.1]	26.1 [26.1 26.2]
3–4	29.8 [29.6 29.9]	30.1 [30.0 30.2]	28.9 [28.8 29.0]	29.3 [29.1 29.4]	28.7 [28.5 28.8]	29.3 [29.2 29.4]	29.5 [29.4 29.5]	29.9 [29.9 29.9]	29.2 [29.1 29.2]	29.6 [29.5 29.6]
5 +	34.2 [34.1 34.3]	34.1 [33.9 34.2]	33.4 [33.2 33.5]	33.3 [33.1 33.4]	33.7 [33.5 33.8]	33.7 [33.5 33.8]	33.9 [33.9 34.0]	33.9 [33.9 34.0]	33.3 [33.2 33.4]	33.5 [33.4 33.6]
Age at first sex										
< 15 Years					26.9 [26.7 27.1]	25.9 [25.7 26.2]	26.0 [25.9 26.1]	26.4 [26.3 26.5]	24.5 [24.5 24.6]	25.3 [25.2 25.4]
15–17 Years					27.7 [27.6 27.8]	27.8 [27.6 27.9]	27.3 [27.3 27.4]	27.8 [27.7 27.8]	26.0 [26.0 26.1]	26.8 [26.7 26.8]
> = 18 Years					29.6 [29.5 29.7]	29.8 [29.6 29.9]	29.5 [29.4 29.5]	29.1 [29.1 29.2]	28.8 [28.8 28.8]	28.1 [28.1 28.2]
Family structure										
Nuclear					28.8 [28.7 28.9]	28.7 [28.6 28.7]	28.7 [28.6 28.7]	28.6 [28.5 28.6]	27.8 [27.8 27.9]	27.6 [27.5 27.6]
Non-nuclear					27.9 [27.8 28.0]	28.2 [28.1 28.3]	27.4 [27.3 27.4]	28.1 [28.1 28.1]	26.7 [26.6 26.7]	27.1 [27.1 27.2]
Prior relationship with husband										
No							28.2 [28.2 28.2]	28.4 [28.4 28.4]	27.4 [27.4 27.5]	27.4 [27.4 27.4]
Yes							27.4 [27.3 27.5]	28.3 [28.2 28.4]	26.6 [26.5 26.7]	27.4 [27.3 27.4]
Total	30.6 [30.6 30.7]		29.4 [29.3 29.5]		28.4 [28.3 28.5]		28.1 [28.1 28.1]		27.4 [27.3 27.4]	

Estimates were adjusted for the all above-mentioned variables.

Appendix Fig. A2 depicts state-specific predicted mean age at last birth among women aged 40–49 years for the last three survey rounds conducted between 2005 and 2021. The state-specific estimates were adjusted for education, residence, caste, religion, and wealth index. The mean age at last birth ranges from 26.0 years in Tamil Nadu to 32.9 years in Meghalaya during NFHS-III. Thereafter in the last two survey rounds, Andhra Pradesh depicts the lowest and Meghalaya shows the highest value of predicted mean age at last birth. A decreasing pattern for mean age at last birth from 2005–2006 to 2019–2021 is also evident in Fig. A2.

The decomposition analysis model takes into consideration the differences in characteristics (compositional factors) as well as differences caused by the effect of characteristics (Table 5). The overall multivariate decomposition analysis results show that approximately 44% of the overall decrease in the mean age at last birth from the period 1992–2021 was due to differences in characteristics, whereas during the period from 1992 to 2006, it was approximately 60%. Among the compositional factors, the majority decline in age at last birth during NFHS-I and NFHS-V was explained by previous parity (75.2%), followed by contraceptive demand (3.2%) and mass media exposure (1.5%). After controlling for the effects of compositional factors, 56% of the change in mean age at the last birth was due to differences in the effects of characteristics.

**Table 5:** Multivariate decomposition result showing the change in age at Last Birth among reproductive-aged women in India, 1992–2021.

Background Characteristics	1992–1993 to 2005–2006								2005–06 to 2019–21								1992–93 to 2019–21							
	Due to difference in Characteristics (E)				Due to difference in Coefficients (C)				Due to difference in Characteristics (E)				Due to difference in Coefficients (C)				Due to difference in Characteristics (E)				Due to difference in Coefficients (C)			
	Coef	SE	*P* value	Percent contribution	Coef	SE	*P* value	Percent contribution	Coef	SE	*P* value	Percent contribution	Coef	SE	*P* value	Percent contribution	Coef	SE	*P* value	Percent contribution	Coef	SE	*P* value	Percent contribution
Age				**0.5**				**6.2**				** − 4.6**				**8.2**				** − 1.2**				**6.9**
40–44																								
45–49	− 0.012	0.001	0.000	0.5	− 0.141	0.041	0.001	6.2	0.050	0.002	0.000	− 4.6	− 0.088	0.027	0.001	8.2	0.041	0.001	0.000	− 1.2	− 0.232	0.033	0.000	6.9
Education				− **3.8**				**3.3**				**3.6**				− **17.0**				− **3.3**				− **1.3**
No education																								
Primary	0.003	0.000	0.000	− 0.1	− 0.070	0.024	0.003	3.1	0.003	0.000	0.000	− 0.3	0.052	0.016	0.001	− 4.8	0.005	0.001	0.000	− 0.1	− 0.017	0.019	0.380	0.5
Secondary	0.003	0.011	0.797	− 0.1	− 0.010	0.024	0.666	0.5	0.013	0.001	0.000	− 1.2	0.070	0.026	0.008	− 6.5	0.047	0.005	0.000	− 1.4	0.029	0.020	0.153	− 0.9
Higher	0.081	0.006	0.000	− 3.6	0.004	0.009	0.654	− 0.2	− 0.055	0.001	0.000	5.1	0.060	0.012	0.000	− 5.6	0.057	0.001	0.000	− 1.7	0.033	0.008	0.000	− 1.0
Mass media				**4.1**				**10.0**				− **2.3**				− **12.8**				**1.5**				**3.3**
No																								
Any	− 0.093	0.021	0.000	4.1	− 0.229	0.194	0.238	10.0	0.025	0.002	0.000	− 2.3	0.138	0.166	0.406	− 12.8	− 0.050	0.005	0.000	1.5	− 0.109	0.145	0.452	3.3
Age at marriage				− **15.3**				− **21.9**				− **41.2**				− **44.7**				− **25.6**				− **27.2**
< 15 Years																								
15–17 Years	− 0.004	0.000	0.000	0.2	0.096	0.036	0.008	− 4.2	− 0.019	0.001	0.000	1.8	0.117	0.026	0.000	− 10.8	− 0.025	0.001	0.000	0.7	0.215	0.029	0.000	− 6.4
≥ 18 Years	0.353	0.007	0.000	− 15.5	0.404	0.047	0.000	− 17.7	0.462	0.003	0.000	− 43.0	0.364	0.040	0.000	− 33.8	0.884	0.006	0.000	− 26.4	0.699	0.038	0.000	− 20.8
Previous parity				**64.0**				**46.6**				**96.6**				− **32.2**				**75.2**				**20.6**
Zero																								
1–2	0.418	0.024	0.000	− 18.3	− 0.334	0.063	0.000	14.6	0.351	0.006	0.000	− 32.7	0.083	0.061	0.177	− 7.7	0.804	0.014	0.000	− 24.0	− 0.286	0.055	0.000	8.5
3–4	− 0.157	0.004	0.000	6.9	− 0.439	0.084	0.000	19.3	− 0.345	0.003	0.000	32.1	0.223	0.045	0.000	− 20.7	− 0.525	0.004	0.000	15.6	− 0.194	0.073	0.008	5.8
5 And more	− 1.720	0.026	0.000	75.4	− 0.289	0.096	0.003	12.7	− 1.044	0.007	0.000	97.1	0.040	0.031	0.185	− 3.8	− 2.803	0.016	0.000	83.5	− 0.210	0.082	0.010	6.2
Contraceptive demand				**5.4**				− **71.9**				**5.0**				− **107.7**				**3.2**				− **81.2**
Unmet need																								
Met need	− 0.317	0.013	0.000	13.9	0.908	0.108	0.000	− 39.8	− 0.144	0.005	0.000	13.4	0.924	0.092	0.000	− 86.0	− 0.315	0.010	0.000	9.4	1.686	0.086	0.000	− 50.2
No demand	0.193	0.013	0.000	− 8.4	0.731	0.079	0.000	− 32.1	0.090	0.004	0.000	− 8.4	0.234	0.043	0.000	− 21.7	0.208	0.010	0.000	− 6.2	1.041	0.063	0.000	− 31.0
Caste				**0.3**				− **12.9**				− **0.3**				**11.6**				− **0.2**				− **4.7**
SC																								
ST	0.000	0.000	0.679	0.0	0.119	0.024	0.000	− 5.2	− 0.003	0.003	0.209	0.3	0.000	0.016	0.984	0.0	− 0.003	0.003	0.209	0.1	0.119	0.020	0.000	− 3.5
Others	− 0.007	0.005	0.180	0.3	0.175	0.110	0.111	− 7.7	0.006	0.003	0.030	− 0.6	− 0.125	0.062	0.046	11.6	0.010	0.005	0.030	− 0.3	0.040	0.092	0.664	− 1.2
Religion				− **0.1**				− **2.3**				**1.4**				− **3.3**				**0.3**				− **2.5**
Hindu																								
Muslim	0.004	0.001	0.000	− 0.2	0.004	0.015	0.760	− 0.2	− 0.009	0.000	0.000	0.8	0.044	0.011	0.000	− 4.1	− 0.002	0.000	0.000	0.1	0.046	0.012	0.000	− 1.4
Christian	− 0.003	0.000	0.000	0.1	0.037	0.017	0.032	− 1.6	− 0.007	0.001	0.000	0.6	− 0.017	0.012	0.151	1.6	− 0.009	0.001	0.000	0.3	0.019	0.014	0.162	− 0.6
Others	0.000	0.000	0.090	0.0	0.012	0.011	0.284	− 0.5	0.000	0.000	0.253	0.0	0.009	0.008	0.245	− 0.8	0.000	0.000	0.254	0.0	0.020	0.009	0.021	− 0.6
Wealth index				**4.6**				**8.2**				− **23.8**				− **17.4**				− **5.2**				**0.6**
Poorest																								
Poorer	0.003	0.000	0.000	− 0.1	− 0.070	0.025	0.005	3.1	− 0.064	0.003	0.000	6.0	− 0.001	0.017	0.944	0.1	− 0.061	0.003	0.000	1.8	− 0.071	0.019	0.000	2.1
Middle	0.004	0.000	0.000	− 0.2	− 0.065	0.032	0.046	2.8	− 0.043	0.001	0.000	4.0	− 0.001	0.023	0.956	0.1	− 0.039	0.001	0.000	1.2	− 0.066	0.025	0.007	2.0
Richer	0.025	0.002	0.000	− 1.1	− 0.062	0.045	0.167	2.7	0.045	0.001	0.000	− 4.2	0.045	0.031	0.147	− 4.2	0.067	0.002	0.000	− 2.0	− 0.014	0.034	0.670	0.4
Richest	− 0.136	0.008	0.000	5.9	0.009	0.065	0.896	− 0.4	0.318	0.008	0.000	− 29.6	0.145	0.054	0.007	− 13.5	0.206	0.005	0.000	− 6.1	0.130	0.052	0.012	− 3.9
Residence				**0.9**				− **19.0**				**4.6**				**56.5**				**0.5**				**6.8**
Urban																								
Rural	-0.021	0.010	0.029	0.9	0.434	0.199	0.029	-19.0	-0.050	0.006	0.000	4.6	-0.607	0.119	0.000	56.5	-0.017	0.002	0.000	0.5	-0.227	0.166	0.170	6.8
State regions				− **0.2**				− **9.6**				− **3.6**				**49.6**				− **1.6**				**9.7**
East																								
West	0.000	0.001	0.962	0.0	0.061	0.025	0.015	− 2.7	0.024	0.001	0.000	− 2.2	− 0.129	0.016	0.000	12.0	0.036	0.002	0.000	− 1.1	− 0.080	0.020	0.000	2.4
North	− 0.015	0.005	0.001	0.6	0.013	0.037	0.720	− 0.6	− 0.004	0.000	0.000	0.4	− 0.135	0.021	0.000	12.6	0.012	0.001	0.000	− 0.4	− 0.153	0.030	0.000	4.5
South	− 0.014	0.002	0.000	0.6	0.020	0.030	0.511	− 0.9	0.018	0.001	0.000	− 1.7	− 0.094	0.022	0.000	8.8	− 0.004	0.000	0.000	0.1	− 0.067	0.025	0.007	2.0
Central	− 0.005	0.001	0.000	0.2	0.024	0.030	0.416	− 1.1	0.007	0.002	0.000	− 0.7	− 0.089	0.020	0.000	8.3	0.006	0.001	0.000	− 0.2	− 0.068	0.024	0.005	2.0
Northeast	0.038	0.006	0.000	− 1.6	0.101	0.021	0.000	− 4.4	− 0.006	0.001	0.000	0.5	− 0.086	0.020	0.000	8.0	0.005	0.001	0.000	− 0.1	0.043	0.017	0.014	− 1.3
Constant					− 2.348	0.463	0.000	102.9					− 1.870	0.303	0.000	173.9					− 4.218	0.378	0.000	125.7
Total				**60.5**				**39.5**				**35.3**				**64.7**				**43.6**				**56.4**

Overall contribution for each characteristics are in [bold].

## Discussion

The ages at which women start and end their childbearing are the key demographic factors that influence fertility. Therefore, the age at last birth becomes an important dimension of the overall fertility level. Our results suggest that there has been a substantial decline in the median age at last birth among Indian women. Previously, women had a wider reproductive period as they used to start childbearing at an early age and continued childbearing through later years of life. Our findings show that, in recent years India is witnessing a shortened reproductive period as women are ending their childbearing at an early age. Moreover, there has been an increase in the proportion of women completing childbearing in the early years of life instead of childbearing through later ages. The results from the multivariate decomposition analysis showed that during 1992–1993 to 2005–2006, the contribution of background characteristics in the change of age at last birth was 60%. Despite the subsequent increase in the proportion of women ending childbearing early, it is to be noted that around one-fourth of the women are bearing the last child after the age of 30 years, and the median age at last birth among women aged 45–49 has declined to 27 years. A study conducted in the United States reported the median age at the last birth to be 31 years among all mothers aged 45–49^38^. In our study, we find that the median age at last birth has declined by five years during the last 3 decades.

Findings show that the age at cessation of childbearing was earlier among respondents from the recent survey than among respondents in the first and second rounds of the survey. This finding is in line with trends observed in Quebec^20^. This stopping behavior might have been due to increased awareness regarding fertility control measures and reduced infant mortality since the age at last birth is associated with fertility reductions and worldwide fertility decline. It was observed that around 17% of eligible women are giving birth after the age of 35 years. Women bearing children after the age of 35 years are twice likely to have miscarriages^14^. The age of 40 and older is in itself identified as an independent risk factor for gestational diabetes, placental abruption, perinatal mortality, and hypertensive complications^7,14^. Studies that have looked into the effect of advanced maternal age on neonatal complication rates show hypertensive disorders, longer NICU admission, preterm labor and intrauterine growth restriction^39^. Researchers highlight that giving birth after the age of 35 has a selective impact on long-term health status among women over the age of 35 years^40^. Also, women aged 35–39 are reported to have an increased risk for fetal/neonatal congenital anomalies, cesarean delivery, and gestational diabetes^7,14^.

The median age at last birth varied significantly across the survey rounds with the lowest median age at last birth observed in the south and the highest in the north-eastern region of India. Hierarchical clustered eat map revealed that a number of states are now closer in terms of age at last birth. The variation could be possibly due to differences in the knowledge, education, and cultural practices that might influence women's reproductive behavior. We found that a low proportion of women in the 90 s reproduced until later ages; however, there has been an increase in the proportion of women stopping their childbearing at an early age. A thorough investigation into human behavioral ecology suggests that the shift in the adaptive motivations has led to fertility decline and delayed reproductive life span. Since reproductive behaviors are affected by social transmissions^41^, factors such as parity and education also play an important role in contraception usage as experienced by countries such as Ethiopia and Poland^41,42^. As evident from the results obtained from the decomposition analysis, factors such as mass media and previous parity reflect the positive contribution in the behavior of decreasing age at last birth whereas along with compositional change in the characteristics between 2005–2006 and 2019–2021.

It was observed that hazards of having lower age at last birth were significantly higher among those with mass media exposure, non-nuclear household, those who were related to their husband before marriage, and without an unmet need than their respective counterparts. Studies based on labor-force participation and fertility indicate that women with long work histories bear children later than those without little or no work experience ^43,44^. This study reinforces the importance of individual-level characteristics affecting the age at last birth. Moreover, delayed childbearing has been documented to positively impact the survival chances in the post-reproductive life of women^45^. Our finding shows that highly educated women were less likely to have last motherhood in the early years of their reproductive period than women who did not receive any formal education in the past years in line with previous studies. Researchers believe that women shift their reproductive behavior in response to educational attainments^37^.

Results obtained from the MCA table also indicated an upward shift in the age at last birth with improved educational attainment which is possibly an indication of uptake of contraception. In contrast to our findings, results from a study conducted in southwest China found that the highest education level was unlikely to interfere with the reproductive career^46^. However, findings show that intermediate education categories (primary, secondary) have a lower age at last birth compared to those without education. Pressure to have a child immediately after marriage and little control over their reproductive rights among uneducated women leads to higher rates of unintended pregnancies and continue childbearing at higher ages. In contrast, women with lower levels of education have better access to and knowledge about contraception. Moreover, resolving unintended pregnancies through abortion and lowering the desired number of children and motivation to enter the labor force to earn can contribute towards reduced age at last birth. Further, in the unadjusted predicted age at last birth across educational categories, the lower three categories (no, primary, secondary education) saw a reduction of approximately 2 years or more however the reduction was not the same in the highest group. Perceptions about the biological clock of women’s bodies, reduction in female fecundity, and existing societal pressure are also likely to impact age at the last birth. It has been shown that women with higher education embark on parenthood later in life^47^. Further, the impact of parenthood status in the labor market and the time available for work and career may have consequences on the age at last birth^48^.

Moreover, in our study age at last birth was found higher among women with higher parity, those who got married before 15 years of age, and Muslim women. Women who marry at an early age tend to start childbearing at an early age and have many children in the absence of control over sexual and reproductive rights and their own bodily rights. Researchers from Bangladesh reported that children born to women married as children have increased mortality rates^49^. In contrast, children born to mothers who were naturally fertile until a relatively high age lived significantly longer^15^ and thus impacting the child health scenario in the country. One of the reasons for the delayed childbearing could be the changes in reproductive behavior due to the expansion of family planning options and the widespread use of birth control measures.

A wide variation in the predicted mean age at the last birth was observed across states. Studies have shown differences in fertility behavior across regions attributable to cultural shifts that have influenced reproductive decision-making^37^. In addition, researchers are of the view that matri- or patri-lineal institutions may play a role in reproductive timing^37^. There is also evidence that women stop childbearing after their desired family size is achieved^3^. Access to contraception reduces the risk of unintended pregnancies and provides women access to stop or space childbearing at the desired time. Moreover, the desired notion of the ideal two-child norm in the recent time might impact the reduced age at the last birth in the past. The usage of contraception could have been one of the pathways to achieve it sooner. Those with met contraception demand have higher age at last birth than those with unmet contraception demand^50^. In accordance with the above-presented significance of contraception in the reduction of age at last birth, our findings showed a major contribution of contraception demand in reducing the age at last birth (results from decomposition analysis). Contraception access reduces the chances of unintentional pregnancies to a greater extent and thus reducing the age at the last birth. As such access to quality contraceptive services enables women to control/stop childbearing which is otherwise in case of unmet contraception demand.

Based on the findings of this study, it is recommended that efforts to educate women and couples about the importance of family planning and the use of contraception should be increased. Additionally, information about the potential health risks associated with early and late childbearing should be provided that can help individuals make informed decisions about their reproductive choices. Ensuring easy access to a wide range of contraception methods, including long-acting reversible contraceptives (LARCs) and modern methods will help individuals and couples effectively plan their pregnancies and avoid unintended pregnancies. Additionally, promoting regular health check-ups, providing counseling on reproductive health, and addressing the specific needs and risks associated with different age groups and reproductive health services should be strengthened. Tailored education campaigns, improved access to contraception, and support services to empower high-risk women can be beneficial, as developing targeted interventions to address their specific needs and making informed decisions about their reproductive health.

## Conclusion

Given the wide importance and contribution of age at the last birth in the context of fertility decline, this study aimed to assess the trends and patterns of age at the last birth in India. Our results indicated that the age at cessation of childbearing among Indian women has decreased in recent years. Education and mass media exposure was found to a significant variable explaining the variations in age at the last birth in the Indian context. Although the proportion of women having children in later years of life has reduced, the reasons underlying this trend in childbearing are complex, and the factors underlying need to be studied. This study shows that there has been a change in the child-bearing pattern and highlights the need to address the outcomes associated with advancing maternal age. Although there exists the need to delay age at first childbirth, the median age at last childbirth also plays an important role in women’s own and their children's health and survival. Therefore, it is important to address the healthcare needs of those delaying their childbirth to a certain extent.

## Supplementary Information

Supplementary Information.

## Data Availability

The datasets generated and/or analyzed during the current study are available in the DHS (Demographic Health Surveys) program repository which is freely available and can be accessed using https://dhsprogram.com/data/dataset_admin/index.cfm.
