# Supplementary material for: Changes in age at last birth and its determinants in India
Source: Sci Rep. 2023 Jun 27;13:10450. doi: 10.1038/s41598-023-37370-z (PMC10300096; doi:10.1038/s41598-023-37370-z)
Supplement: Supplementary file 1 — Supplementary Information. [file 41598_2023_37370_MOESM1_ESM.docx]

Appendix

**Changes in age at last birth and its determinants in India**

**Figure A1:** State specific hierarchical clustered heat map of probability of last birth not yet happened by age among women aged 40-49 years.

| I.  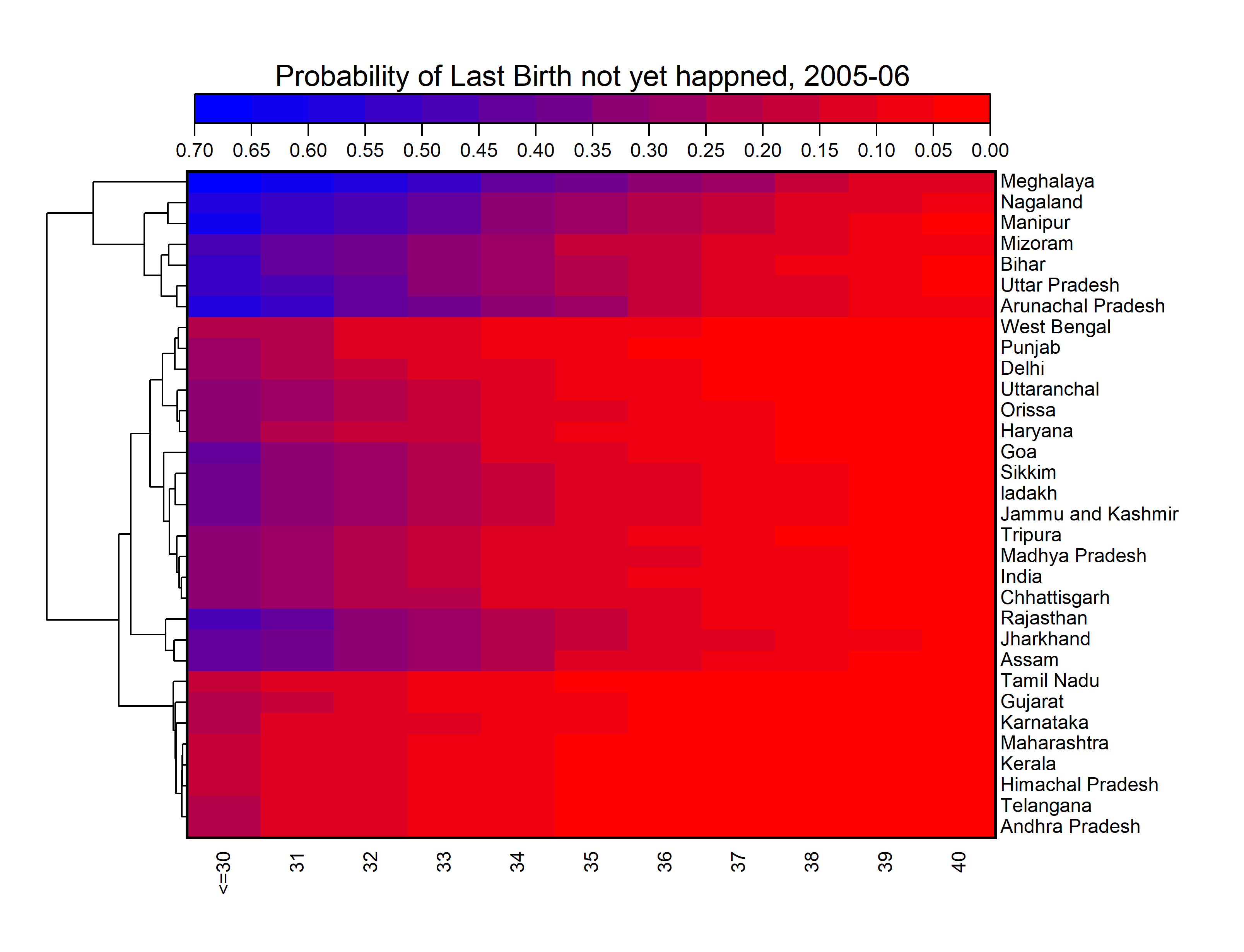 |
| --- |
| II.  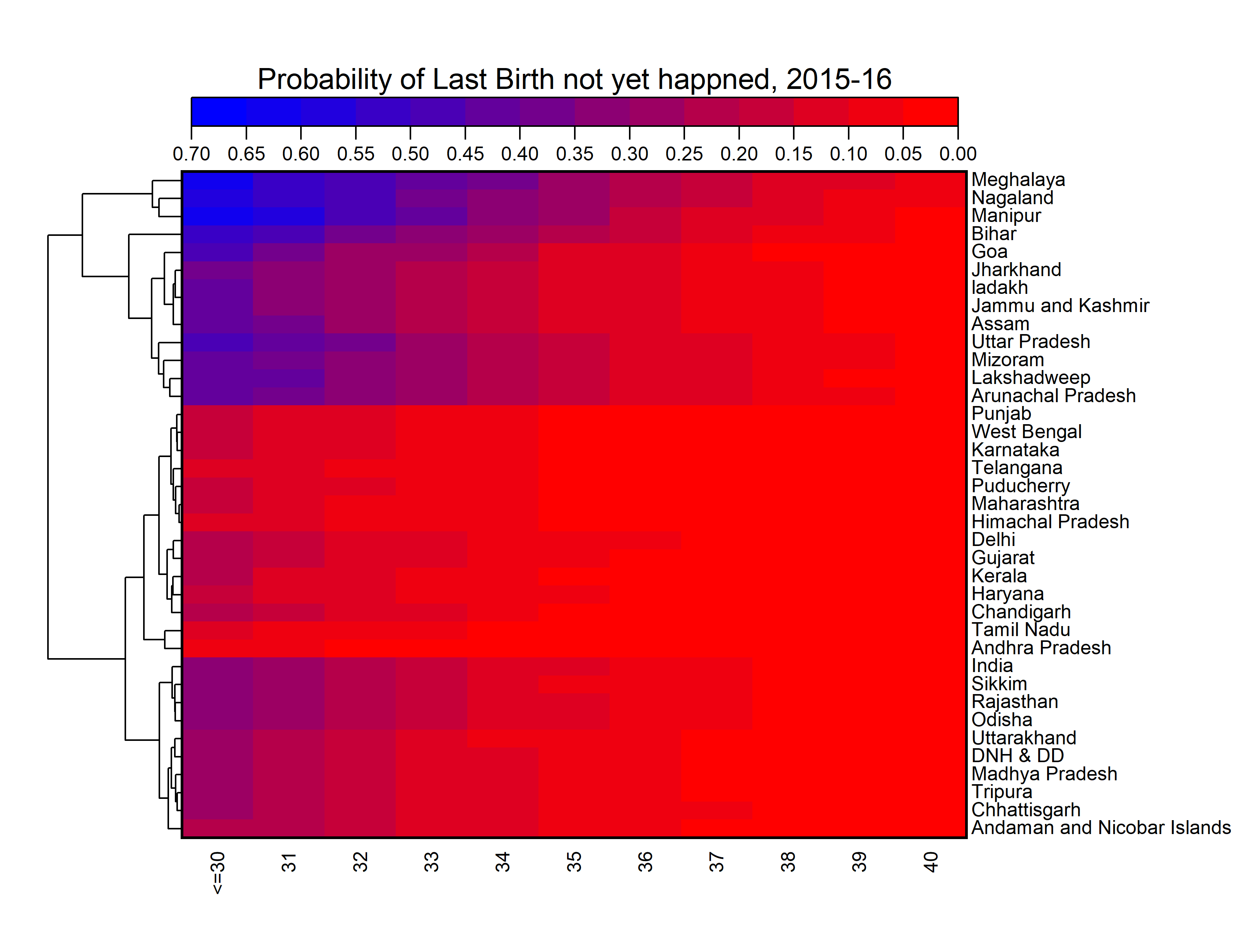 |
| III.  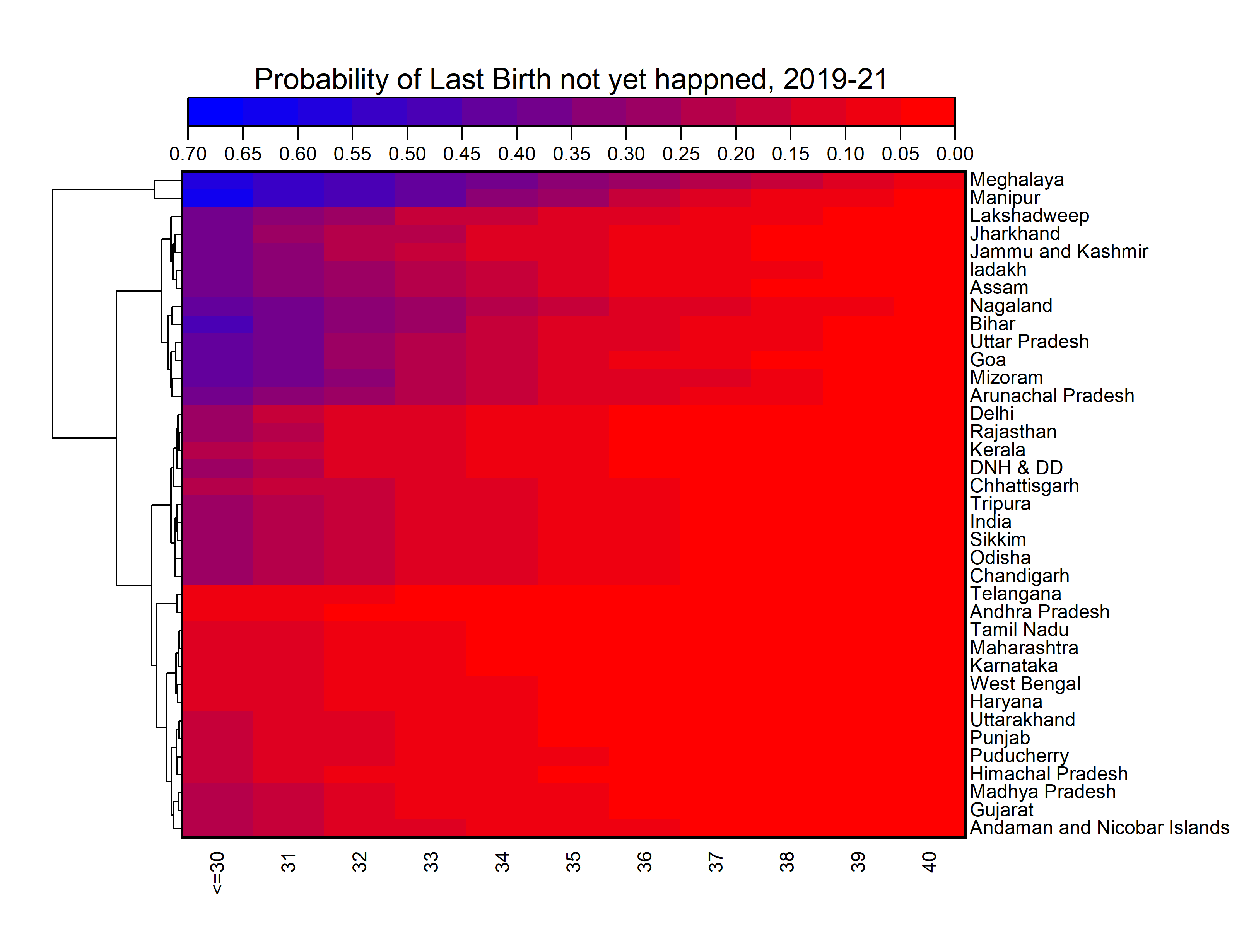 |

**Table B1:** Cox Proportional Hazard Model predicting women risk of Last Birth by various demographic characteristics, 2019-21.

| **Characteristics** | **AHR [95% CI]** | | |
| --- | --- | --- | --- |
| **Individual Characteristics** | **Model 1** | **Model 2** | **Model 3** |
| **Current Age** |  |  |  |
| 40-44 (Ref) |  |  |  |
| 45-49 | 0.86*** [0.85,0.86] | 0.84*** [0.84,0.85] | 0.83*** [0.82,0.83] |
| **Education** |  |  |  |
| No Education(Ref) |  |  |  |
| Primary | 1.13*** [1.12,1.15] | 1.09*** [1.08,1.11] | 1.09*** [1.08,1.10] |
| Secondary | 1.06*** [1.05,1.07] | 0.98*** [0.97,0.99] | 0.95*** [0.94,0.96] |
| Higher | 0.70*** [0.69,0.71] | 0.61*** [0.60,0.62] | 0.59*** [0.58,0.60] |
| **Mass Media Exposure** |  |  |  |
| No(Ref) |  |  |  |
| Any | 1.27*** [1.26,1.28] | 1.12*** [1.11,1.13] | 1.10*** [1.08,1.11] |
| **Age at First Marriage** |  |  |  |
| <15 years |  |  |  |
| 15-17 years | 0.87*** [0.86,0.87] | 0.87*** [0.87,0.88] | 0.86*** [0.85,0.87] |
| >=18 years | 0.43*** [0.42,0.43] | 0.44*** [0.44,0.44] | 0.43*** [0.42,0.43] |
| **Previous Parity** |  |  |  |
| Zero |  |  |  |
| 1-2 | 0.70*** [0.69,0.71] | 0.72*** [0.71,0.73] | 0.73*** [0.72,0.74] |
| 3-4 | 0.36*** [0.35,0.36] | 0.39*** [0.38,0.39] | 0.40*** [0.40,0.41] |
| 5 and More | 0.18*** [0.17,0.18] | 0.19*** [0.19,0.20] | 0.21*** [0.21,0.22] |
| **Contraceptive Demand** |  |  |  |
| Unmet need |  |  |  |
| Met need | 1.81*** [1.78,1.84] | 1.74*** [1.71,1.77] | 1.70*** [1.67,1.73] |
| No demand | 1.46*** [1.43,1.48] | 1.46*** [1.43,1.48] | 1.46*** [1.44,1.49] |
| **Household Characteristics** |  |  |  |
| **Residence** |  |  |  |
| Urban(Ref) |  |  |  |
| Rural |  | 1.06*** [1.05,1.07] | 1.06*** [1.05,1.07] |
| **Caste** |  |  |  |
| SC (Ref) |  |  |  |
| ST |  | 1.03*** [1.01,1.04] | 1.02** [1.01,1.04] |
| Others |  | 1.01 [1.00,1.01] | 1.03*** [1.02,1.04] |
| **Religion** |  |  |  |
| Hindu(Ref) |  |  |  |
| Muslim |  | 0.84*** [0.83,0.85] | 0.82*** [0.81,0.83] |
| Christian |  | 0.86*** [0.84,0.87] | 0.86*** [0.85,0.88] |
| Others |  | 1.01 [1.00,1.03] | 1.02 [1.00,1.03] |
| **Wealth Index** |  |  |  |
| Poorest(Ref) |  |  |  |
| Poor |  | 1.19*** [1.17,1.20] | 1.20*** [1.19,1.22] |
| Middle |  | 1.32*** [1.31,1.34] | 1.37*** [1.35,1.39] |
| Richer |  | 1.42*** [1.40,1.44] | 1.50*** [1.48,1.52] |
| Richest |  | 1.53*** [1.51,1.55] | 1.66*** [1.63,1.68] |
| **State Regions** |  |  |  |
| East(Ref) |  |  |  |
| West |  | 1.16*** [1.14,1.18] | 1.18*** [1.17,1.20] |
| North |  | 1.02** [1.01,1.03] | 1.02** [1.01,1.03] |
| South |  | 1.25*** [1.23,1.26] | 1.27*** [1.26,1.29] |
| Central |  | 0.96*** [0.94,0.97] | 0.94*** [0.93,0.95] |
| Northeast |  | 0.93*** [0.91,0.94] | 0.94*** [0.92,0.95] |
| **Year of Survey** |  |  |  |
| 1992-93 (Ref) |  |  |  |
| 1998-99 |  |  | 1.18*** [1.16,1.21] |
| 2005-06 |  |  | 1.26*** [1.24,1.29] |
| 2015-16 |  |  | 1.42*** [1.40,1.45] |
| 2019-21 |  |  | 1.50*** [1.47,1.53] |
| **Log Likelihood** | **-3784559.3** | **-3620934.5** | **-3619580.8** |
| **Note:** * p<0.05, ** p<0.01, *** p<0.001, AHR: Adjusted hazard ratio, CI: Confidence Interval | |  |  |

**Figure A2:** State specific predicted mean age at Last Birth among women aged 40-49 years by survey rounds.

| A.  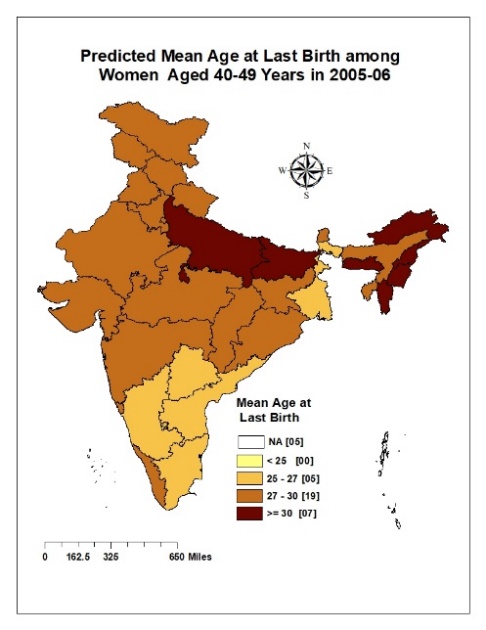 | B.  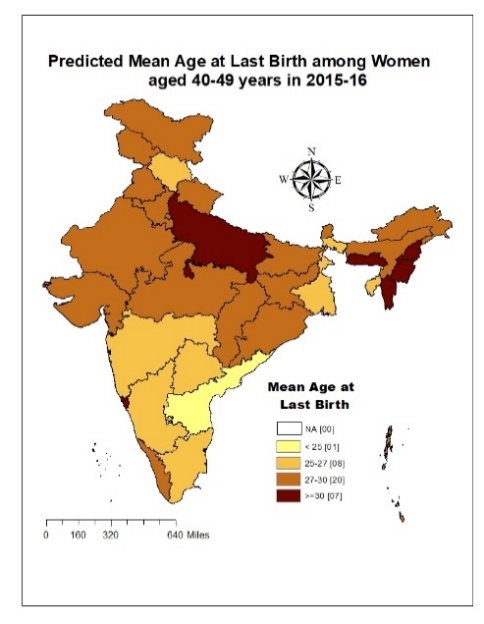 | C.  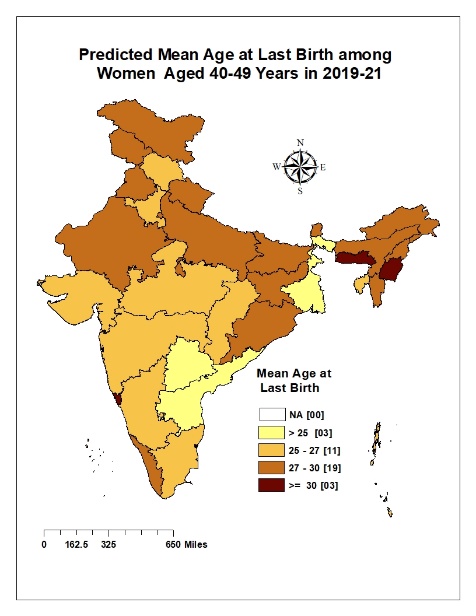 |
| --- | --- | --- |

Notes: A, B and C are predicted mean age last birth estimates for the year 2005-06, 2015-16 and 2019-21 respectively, Estimates are adjusted for education, residence, caste, religion and wealth index.
